# Hydration for Adult Patients with Nephrolithiasis: Specificities and Current Recommendations

**DOI:** 10.3390/nu15234885

**Published:** 2023-11-22

**Authors:** Marie Courbebaisse, Simon Travers, Elise Bouderlique, Arthur Michon-Colin, Michel Daudon, Aurélie De Mul, Laura Poli, Stéphanie Baron, Caroline Prot-Bertoye

**Affiliations:** 1 Faculté de Médecine, Université Paris Cité, F-75006 Paris, France; 2 Institut Necker Enfants Malades, Inserm U1151, F-75015 Paris, France; 3 Physiology—Functional Explorations Department, Georges Pompidou European Hospital, AP-HP, F-75015 Paris, Francecaroline.bertoye@aphp.fr (C.P.-B.); 4 Centre de Référence des Maladies Rénales Héréditaires de l’Enfant et de l’Adulte (MARHEA), F-75015 Paris, France; 5 Centre de Référence des Maladies Rares du Calcium et du Phosphate, F-75015 Paris, France; 6 Équipe Biologie, Lip(Sys)2, EA7357, UFR de Pharmacie, Université Paris-Saclay, F-91400 Orsay, France; 7 Clinical Chemistry Department, Georges Pompidou European Hospital, AP-HP, F-75015 Paris, France; 8 Department of Multidisciplinary Functional Explorations, Tenon Hospital, AP-HP, F-75019 Paris, France; 9 Centre de Référence des Maladies Rares du Calcium et du Phosphate, Filière Maladies Rares OSCAR, Hôpital Femme Mère Enfant, Hospices Civils de Lyon, F-69500 Bron, France; 10 Centre de Recherche des Cordeliers, INSERM, Sorbonne Université, Université Paris Cité, F-75006 Paris, France; 11 CNRS ERL 8228—Laboratoire de Physiologie Rénale et Tubulopathies, F-75006 Paris, France

**Keywords:** nephrolithiasis, hydration, water, prevention

## Abstract

Nephrolithiasis affects around 10% of the population and is frequently associated with impaired dietary factors. The first one is insufficient fluid intake inducing reduced urine volume, urine supersaturation, and subsequently urinary lithiasis. Kidneys regulate 24 h urine volume, which, under physiological conditions, approximately reflects daily fluid intake. The aim of this study is to synthesize and highlight the role of hydration in the treatment of nephrolithiasis. Increasing fluid intake has a preventive effect on the risk of developing a first kidney stone (primary prevention) and also decreases the risk of stone recurrence (secondary prevention). Current guidelines recommend increasing fluid intake to at least at 2.5 L/day to prevent stone formation, and even to 3.5–4 L in some severe forms of nephrolithiasis (primary or enteric hyperoxaluria or cystinuria). Fluid intake must also be balanced between day and night, to avoid urinary supersaturation during the night. Patients should be informed and supported in this difficult process of increasing urine dilution, with practical ways and daily routines to increase their fluid intake. The liquid of choice is water, which should be chosen depending on its composition (such as calcium, bicarbonate, or magnesium content). Finally, some additional advice has to be given to avoid certain beverages such as those containing fructose or phosphoric acid, which are susceptible to increase the risk of nephrolithiasis.

## 1. Introduction

Kidney stone disease is a global health burden as it affects around 10% of the population, with an increase in its prevalence and in its incidence over the past few decades [1,2,3]. Its annual cost exceeds 2 billion USD in the USA [4]. In the absence of a personalized preventive medical treatment, recurrence of renal calculi at 10 years is high, around 30 to 50% [2,5]. Kidney stones can range from an asymptomatic incidental finding in imaging evaluation of other indications to a painful ureteral obstruction that can be complicated by a urinary tract infection. In addition, nephrolithiasis may be associated with the development of chronic kidney disease [6,7,8].

## 2. Diet and Lithogenesis Mechanisms

**Figure 1:**
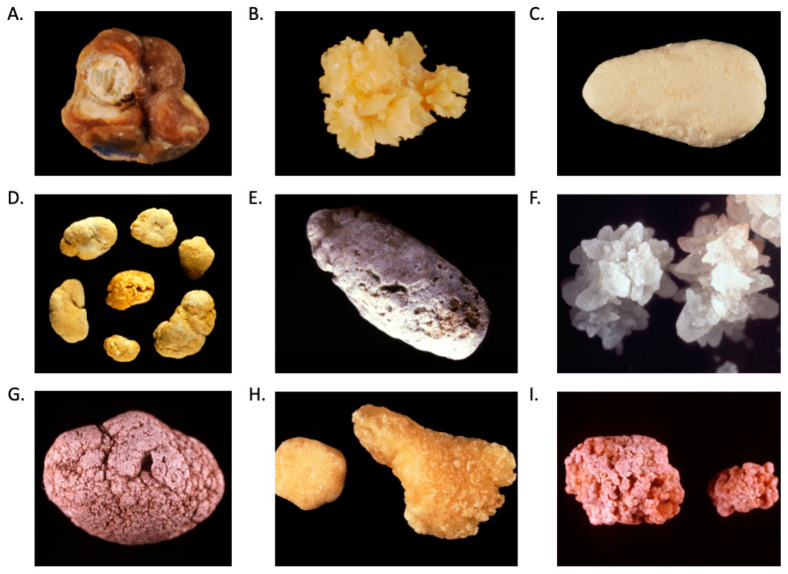
Kidney stones subtypes. (**A**) Calcium oxalate monohydrate (Whewellite), (**B**) calcium oxalate dihydrate (Weddellite), (**C**) calcium phosphate (carbapatite), (**D**) uric acid, (**E**) ammonium urate, (**F**) struvite, (**G**) dihydroxyadenine, (**H**) cystine, (**I**) drug (N-acetylsulfadiazine).

Kidney stones are heterogeneous (Figure 1). Calcium-containing stones are the most common type of kidney stones, accounting for more than 80% of cases, with most being composed of calcium oxalate or more rarely of calcium phosphate [5,9,10] (Figure 1). Uric acid comprises 5% to 10% of all stones, struvite 1% to 5%, and rare stone (e.g., cystine, ammonium urate, drug, dihydroxyadenine) 3% or less [9,10] (Figure 1).

The different types of stones find their origin according to the underlying pathologies and several superadded lithogenesis mechanisms [11]. Stone formation is a multifactorial process that can be associated with:

- Anatomical abnormalities, e.g., medullary sponge kidney, ureteropelvic junction obstruction, horseshoe kidney [12],
- Rare inherited monogenic metabolic disorders [13], e.g., cystinuria characterized by a defective reabsorption of cystine in the renal proximal tubule [14], primary hyperoxaluria, a group of disorders of glyoxylate metabolism that cause the overproduction of endogenous oxalate [15], 2,8-dihydroxyadeninuria due to adenine phosphoribosyltransferase deficiency [16], autosomal dominant or recessive distal renal tubular acidosis type I associated with impaired acid excretion by intercalated cells in the renal collecting duct [17], Dent disease linked to proximal tubular defects [18], hereditary hypophosphatemic rickets with hypercalciuria characterized by renal phosphate wasting [19,20]),
- General diseases: primary hyperparathyroidism [21], granulomatous diseases such as sarcoidosis [22], gastrointestinal diseases inducing enteric hyperoxaluria like jejunoileal bypass [23], metabolic syndrome [24], and diabetes mellitus [25].

Of note, some drugs can also directly or indirectly induce nephrolithiasis [12]. Poorly soluble medications with high urine excretion are susceptible to crystallize in the urine (e.g., amoxicillin, indinavir) and some treatments may also indirectly impair urine composition (e.g., acetazolamide, which dramatically increases urinary pH) [26]. Finally, chronic or recurrent urinary tract infections with urease-producing microorganisms inducing ammoniogenesis cause struvite stones [27]. 

Besides these causes, diet is one of the most important factors of lithogenesis. For example, very common calcium oxalate monohydrate (whewellite) subtype Ia stones are primarily related to high consumption of oxalate-rich foods and precursors of oxalate such as hydroxyproline-rich foods, to low calcium intake (thereby increasing oxalate absorption in the gut), and to insufficient water intake [11].

Regardless of the stone type, stone formation involves urine supersaturation, crystal nucleation (in which solute molecules in a solvent begin to cluster), and the growth and aggregation of stone-forming salts such as calcium oxalate, calcium phosphate, or uric acid [28]. Urine supersaturation, which is a necessary condition for the development of stones, is always due to a reduced urine volume and/or to an excess of solutes in the urine. Importantly, whereas it is not always possible to decrease urinary daily excretion of stone precursors, it is nearly always possible to increase daily urine volume, explaining why increasing fluid intake is the cornerstone of the preventive medical treatment for all types of stones.

## 3. Fluid Intake Is the Main Determinant of Urine Volume (Figure 2)

### 3.1. Water Is the Main Constituent of the Human Body

Around 60% and 50% of the body weight in healthy men and women adults, respectively, is composed of water, owing to differences in body fat [29]. About two-thirds of body water is intracellular, whereas one-third of body water is in the extracellular space (divided between the plasma and the interstitial compartments (in a ratio of 1:3)) [29]. Thus, a man weighting 70 kg is composed of about 42 L of water, distributed in intracellular compartments for 28 L, in plasma for 3.5 L, and in interstitial compartments for 10.5 L.

**Figure 2:**
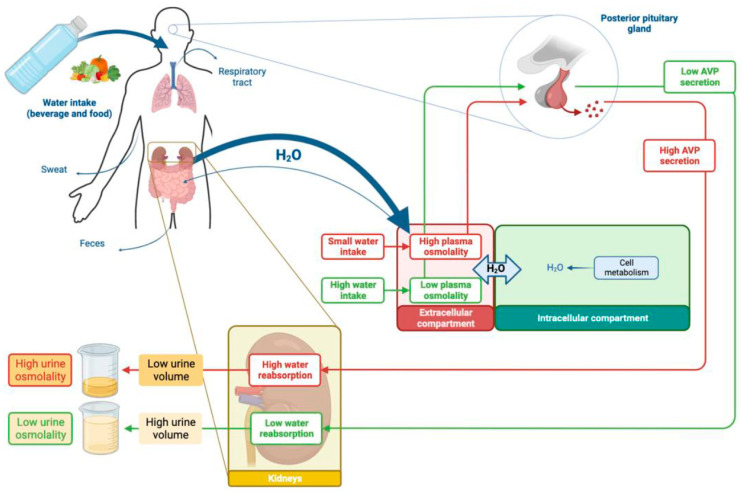
Urine volume and osmolality depend on water intake. In response to water intake, posterior pituitary gland adapts AVP secretion. When water intake is high, plasma osmolality decreases (green arrows), leading to intracellular volume expansion and to inhibition of AVP release. The water reabsorption by the kidney is then minimized, and a large amount of diluted urine is excreted. In case of reduced water intake, plasma osmolality increases (red arrows), leading to a decrease in intracellular volume, to a stimulation of AVP secretion, and finally to a small amount of concentrated urine.

At a steady state, the intracellular volume is in equilibrium with the extracellular space: the intracellular and extracellular osmolalities are equal (about 290 mOsmol/kg H_2_O). Almost all cell membranes express water channels (aquaporin), allowing passive water diffusion according to an osmotic gradient, while cell membranes are relatively impermeable to solutes that must be actively transported. A change in the amount of water in the extracellular compartments without any change in the solute content creates an osmotic gradient between the intracellular and extracellular compartments, resulting in a water flow from one compartment to the other, thereby restoring osmotic equilibrium [29] (Figure 2).

### 3.2. Water Homeostasis Depends on Inflows and Outflows

Water intake mainly comes from beverages and, to a lesser extent, from hydrated foods (vegetables) and from the oxidative metabolism of carbohydrates or fats [30] (Figure 2). Beverage consumption is influenced by thirst in response to a slight increase in plasma osmolality induced by the absorption of salt and other solutes from food, but is also conditioned by social behaviors [29].

Regarding fluid outputs, water output from the kidneys (urine) constitutes the majority of water outflows from the body and is the only one that is regulated [30]. Breathing, perspiration, sweating, and feces constitute other insensitive water outflows from the body, depending on ambient temperature, physical activity, and digestive pathologies. 

In physiological conditions and intermediate climatic situations, insensible losses (from breathing, perspiration, sweating, and feces) are roughly equivalent to insensible intakes (from hydrated foods and metabolism) so that the 24 h urine volume can be approximately considered to reflect the daily fluid intake. 

### 3.3. Kidneys Regulate Water Output and Urine Volume

The kidneys’ urine concentrating and diluting mechanisms are mandatory to maintain a nearly constant blood plasma osmolality without continuous access to water [31]. When water intake is substantial enough to dilute the plasma, more diluted urine is produced to normalize plasma osmolality. In contrast, when water intake is so low that the plasma is concentrated, concentrated urine is produced to restore normal plasma osmolality [31] (Figure 2).

In healthy adult kidneys, the glomeruli filter approximately 180 L per day of ultrafiltrate from the plasma. Most of it is reabsorbed in the tubule, with a massive and non-regulated water reabsorption in the proximal tubule and Henle loop and a more moderate but tightly regulated water reabsorption in the collecting duct. Indeed, the final setting of water reabsorption depends on the water permeability of the collecting duct, enabled by the expression of aquaporin channels controlled by the level of an antidiuretic hormone, plasma arginine vasopressin (AVP) [29,32,33]. 

Under physiological conditions, the most important determinant of AVP secretion by the magnocellular neurons in the hypothalamic neuropituitary tract is the effective osmolality [29]. If the plasma osmolality increases (for example, if water intake is low or if extra renal water losses increase (e.g., diarrhea or sweating), the plasma AVP concentration increases (Figure 2). In turn, AVP activates V2 receptor in the renal collecting duct, leading to an increased production of aquaporin and to its expression at the cell membrane [32,34]. Consequently, the epithelial cells of the collecting duct become permeable to water, allowing water reabsorption from tubular fluid to the blood thanks to the osmotic gradient formed in the renal medulla (the vascular organization favors the accumulation and the concentration of solutes in the renal medulla, generating the osmotic driving force) [29,35]. As a result, urine volume becomes lower and urine concentration higher. Conversely, in the case of high fluid intake associated with low AVP secretion, these renal epithelial cells, as well as their tight junctions, are impermeable to water. Therefore, a larger volume of filtrate is excreted as dilute urine to maintain the plasma osmolality within a narrow range [33].

Furthermore, chronic polyuria interferes with the maintenance of the medullary concentration gradient, decreasing the maximum concentration ability of urine. The extent of the decrease varies proportionally to the urine volume [36].

## 4. Reducing Urine Volume May Increase the Risk of Developing First Kidney Stones

Patients exposed to hot climate or working or performing physical activities at a high ambient temperature have a high incidence of kidney stones [37,38,39,40,41,42,43,44,45,46]. This is likely due, at least in part, to “dehydration”, attributed to heat-induced sweating, thus reducing urine volume and increasing the urine supersaturation of stone-forming salts. An educational program to increase fluid intake in a desert town was associated with a slight increase in urine volume and to a subsequent decrease in the prevalence of urolithiasis [47].

On the other hand, populations that purposefully limit fluid intake to reduce urinary frequency, yielding more concentrated urine, may be at higher risk of developing kidney stones, such as taxi cab drivers or healthcare professionals working in an operating room without proper access to bathroom facilities [46,48,49].

Borghi et al. evaluated the urine volume in patients suffering from their first stone episode. They reported that the urine volume was significantly lower in 199 stone-forming men and women with a first episode of calcium stone than in 101 healthy control subjects (1057 ± 238 vs. 1401 ± 562 mL/day in males, *p* < 0.0001; 990 ± 230 vs. 1239 ± 440 mL/day in females, *p* < 0.0001) [50]. 

Large observational studies including non-stone-forming patients have reported the preventive effect of high fluid intake on kidney stone risk (primary prevention), the risk of kidney stones being inversely correlated with fluid intake. In a prospective observational cohort study of 45,619 men who had no previous history of kidney stones (The National Health Professionals cohort), an inverse association between fluid intake and the risk of kidney stones was found during four years of follow-up [51]. In the quintile consuming over 2500 mL of fluid per day, compared to the quintile consuming less than 1275 ml per day, the relative risk dropped from 1 to 0.71 (95 percent confidence interval 0.52 to 0.97; (*p* = 0.003)) [51]. Taylor et al. reported similar results regarding the beneficial effect of fluid intake in this cohort after a follow-up of 14 years: the relative risk for the men in the highest as compared with the lowest quintile group for fluid intake was 0.71 (95% CI, 0.59 to 0.85; *p* < 0.001 for trend) [52]. This result was also confirmed in large prospective cohorts of women (Nurses’ Health Studies (NHS)) with a follow-up of 8 and 12 years [53,54].

Curhan et al. examined the 24 h urine chemistries in 907 men and women with a history of kidney stone disease and 239 without a history of kidney stone disease who were participants in three large cohort studies (the NHS I (n = 297 cases; n = 99 controls) and II (n = 169 cases, n = 30 controls) and the Health Professionals Follow-up Study (HPFS; n = 341 cases, n = 110 controls)) [55]. The mean age and weight were similar for the cases and controls within the respective cohorts. The mean 24 h urine volume was significantly lower in cases than controls in NHS I (mean (SD) 1.76 L (0.70) vs. 2.15 L (0.91), *p* ≤ 0.01) and HPFS (mean (SD) 1.66 L (0.64) vs. 1.87 L (0.76), *p* ≤ 0.01) [55] but the difference was not significant in NHS II (mean (SD) 1.51 L (0.67) vs. 1.75 L (0.79)), possibly due to a lack of power [55]. 

In a UK-population-based prospective cohort of 439,072 participants over a mean of 6.1 years of follow-up, total fluid intake was inversely associated with the risk of developing a first incident kidney stone [56]. When compared to fluid intake of zero to six glasses per day (approximately 1.2 L), individuals who drank 13 glasses or more (equivalent to ≥2.3 L) had a 50% reduced risk of developing kidney stones [56]. For every additional drink (200 mL) consumed per day of total fluid, the risk of kidney stones declined by 13% (hazard ratio = 0.87, 95% confidence interval 0.85–0.89, *p* value for trend ≤0.001) [56]. 

## 5. Increasing Urine Volume in Stone Formers May Decrease the Risk of Stone Recurrence

The effect of dilution on the crystallization of stone-forming salts in patients with kidney stones and in normal subjects was evaluated by Pak et al. Dilution of urine was achieved in vitro by adding distilled water to urine or in vivo via the intake of distilled water. Urine dilution significantly reduced the urinary activity product ratio of calcium phosphate (brushite), calcium oxalate, and monosodium urate and increased the minimum supersaturation needed to elicit spontaneous nucleation of calcium oxalate, thereby reducing the risk of crystal nucleation [57].

To assess the effect of urine dilution, Borghi et al. examined natural and diluted overnight 8 h urine collections. The overnight 8 h urine was collected from 11.30 p.m. to 07.30 a.m. under normal hydration conditions and after a 500 mL load of oligomineral water administered at 11.30 p.m. in 15 male calcium oxalate stone formers without metabolic urinary anomalies (hypercalciuria, hyperoxaluria, hyperuricosuria, hypocitraturia, and hypomagnesuria) and 12 male controls. The water load reduced the calcium oxalate saturation [58]. It also increased the urine tolerance to oxalate loads (a test consisting of adding increasing amounts of oxalate in order to determine the minimum quantity of oxalate required to start crystallization). Importantly, it did not modify the power of the macromolecules to inhibit calcium oxalate crystallization, despite the concurrent dilution of the calcium crystallization inhibitors induced by the water load [58].

As regards the prevention of recurrence, Hosking et al. reviewed the clinical courses of 108 patients with idiopathic calcium urolithiasis (86.1% of whom had recurrent stones) advised to increase fluid intake to achieve a daily urine output of at least 2500 mL. Patients who had no evidence of stone growth or new stone formation during follow-up had a significantly higher urine volume than those with recurrent stone formation (approximately 2100 mL/day vs. 1700 mL/day (*p* < 0.005)) [59]. In this study, patients were not controlled for other predisposing factors to urinary stone disease such as diet [59].

In a prospective cohort study of idiopathic calcium oxalate stone formers (including 22% (n = 40) first stone formers and 78% (n = 141) multiple stone formers before being referred), Daudon et al. reported a higher mean urine volume in patients without stone recurrence (mean ± SEM: 2260 ± 50 mL/day) compared to patients with stone recurrence (1740 ± 60 mL/day, *p* < 0.0001), each patient having a follow-up of at least 3 years (within a mean follow-up of 6.8 years). The risk of recurrence decreased by 68% for each 1000 mL/day increase in daily urine output [60]. 

Only two randomized trials have been published regarding this topic. In the first one, Sarica et al. randomized 70 stone formers prior to shock wave lithotripsy for calcium oxalate stones into three groups: 25 patients receiving calcium channel blockers, 25 patients undergoing an enforced fluid intake program to achieve a daily urinary output of more than 2500 mL, and 20 patients not receiving any specific medication and/or measure apart from close follow-up after shock wave lithotripsy [61]. The mean follow-up period was 30.4 months (24–36 months). Among patients who became stone-free (12/25, 48%) in the group advised to increase fluid intake, only one patient (1/12, 8.3%) experienced a new stone formation during follow-up, while the figure was 55% (5/9) in patients receiving no specific measure [61]. It should be noted that the characteristics of the subset of participants who become stone-free were not compared to assess that the groups were comparable at baseline, and urine volumes were not reported in this study.

In the second trial, Borghi et al. randomized 220 patients after their first episode of idiopathic calcium stone episode in two different follow-up programs lasting 5 years to either increase water intake with a goal urine volume equal to or greater than 2 L per day without any changes in diet or without any treatment in the control group. There were 199 patients that completed the study, including 99 patients in the intervention group. After the 5-year follow-up period, patients treated with high fluid intake had significantly fewer recurrences (assessed as episodes of renal colic or stone expulsion episodes or at annual imaging) (12.1% of patients) compared with the control group (27% of patients) (*p* = 0.008). In addition, the mean time to relapse was also significantly longer in the treated group than in the control group (38.7 ± 13.2 months vs. 25.1 ± 16.4 months, *p* = 0.016). The high water intake caused a large increase in urine volume associated to a strong reduction in the supersaturation of lithogenous salts throughout the follow-up period. After 5 years, the mean urine volume in the high fluid intake group was 2.6 L/day compared to 1 L/day in the controls, highlighting the protective role of a high fluid intake against recurrence after the first idiopathic calcium stone episode [50]. Of note, the patients included in this study were stone-free after their first lithiasis episode and had no metabolic pathology, excluding patients most at risk of recurrence.

Systematic review and meta-analyses confirmed the significantly reduced risk of kidney stones with high fluid consumption (pooled risk ratios 0.20 (95% confidence interval 0.09–0.44) calculated according to seven observational studies) [62]. 

To our best knowledge, no prospective randomized trial evaluated the effect of urine dilution in high-risk stone formers, such as in the case of kidney anatomical abnormalities or general or genetic diseases associated with stone formation [12]. 

## 6. Increasing Urine Volume in Cystinuric Patients May Decrease Stone Recurrence

The rationale for the amount of urine dilution can be discussed in cystinuric patients. Cystinuria (OMIM 220100), an autosomal recessive disorder, is the most frequent monogenic cause of kidney stones and is responsible for 1% of nephrolithiasis in adults [14]. It affects renal cystine reabsorption. Because of its poor solubility at a typical urine pH lower than 7, excess cystine results in urinary cystine stone recurrent formation. Cystine solubility is <1.05 mmol/L at a pH < 6 and reaches 2.1 mmol/L at a pH > 7.5 [14]. The amount of cystine excreted by cystinuric patients is typically >1.6 mmol/day and usually reaches 2.5–6 mmol/day [14]. Alkaline hyperdiuresis is the cornerstone of the medical preventive therapy [14]. Fluid intake should guarantee a diuresis large enough to maintain a urine cystine concentration below 1 mmol/L [14]. Barbey et al. reported in 27 cystinuric patients that the daily urine volume was significantly higher in patients with arrested stone formation (3151 ± 587 vs. 2446 ± 654 mL/24 h, *p* = 0.006), suggesting that maintaining a daily urine volume of greater than 3 L is essential for therapeutic success [63]. Moreover, the probability of cystine crystalluria that is associated with stone formation among patients with cystinuria [64] increased as urine specific gravity increased from 1.005 to 1.010 g/cm^3^ [65]. It is interesting to underline that if fluid intake is well distributed over the 24 h period, there is an almost linear relationship between morning urine specific gravity and 24 h urine volume. Moreover, a morning specific gravity of 1.005 g/cm^3^ corresponds to a 3 L/24 h diuresis [66], reinforcing the concordance between the two abovementioned targets. 

## 7. Current Guidelines Recommend Increasing Fluid Intake to Prevent Stone Formation

**Figure 3:**
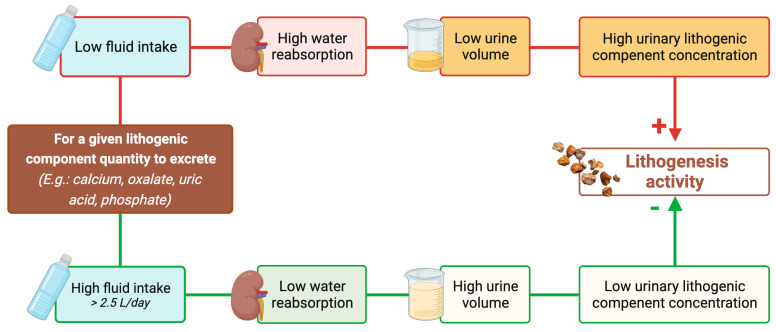
How fluid intake is linked to lithogenesis activity. Urine volume depends on fluid intake and the more a lithogenic component is diluted in urine, the less it can lead to kidney stone formation. Therefore, only high fluid intake (bottom part) ensures adequate urine volume and dilution with low concentration of lithogenic components, preventing stone recurrence.

Given the risk of recurrence, of complications of stone disease, and the high cost of surgical intervention, a medical prophylactic program to reduce stone occurrence is essential. All stone formers should normalize their dietary habits. They are advised to have a diet rich in vegetables and fiber with a normal calcium content and limited content of NaCl and animal protein [12,67,68]. In addition to these recommendations, patients are encouraged to maintain a generous fluid intake [12] to increase the urine flow rate in order to lower the concentration of stone-forming constituents such as calcium, oxalate, phosphate, and uric acid with a subsequent reduction in the supersaturation of stone-forming salts (Figure 3).

Current international guidelines include general recommendations regarding the fluid intake for preventing stone recurrences. The Canadian Urological Association and American Urological Association recommend that all stone formers should be counseled to achieve a daily urine output of 2.5 L [69,70]. The European Association of Urology mentions a fluid intake of 2.5–3 L to promote a urine volume of at least 2.0–2.5 L each day [12] (Table 1).

In high-risk stone formers, such as in patients with cystinuria or primary hyperoxaluria, it is recommended to maintain a high fluid intake of 3.5–4 L, ensuring a daily urine output of at least 3 L each day (Table 1).

Urine produced during the night is the most concentrated and therefore carries the highest risk of supersaturation and crystal formation. For this reason, fluid intake should be well distributed over day and night [12]. Patients should be advised to spread their consumption throughout the day, not forgetting before bedtime and each time they wake at night. 

Fluid intake should be higher, including the replacement of extra renal fluid outflow, in case of excessive sweating due to a high physical activity level or high ambient temperature or in the case of digestive losses (diarrhea) [12].

Equally, 24 h urine collection is essential for diagnosing and following stone patients [72]. The crystallogen risk thresholds above which, for promoters, or below which, for inhibitors, crystallization is significantly more frequent have been defined (Table 2) [73]. The therapeutic objective is to bring the urine into an area where the crystallogenic risk is reduced [73]. For example, the urine dilution should lower the urinary concentration of oxalate and calcium below the threshold values of 0.31 mmol/L for oxalate and 3.8 mmol/L for calcium [60,74]. However, calculating the relative supersaturation values using computer programs provides a better indication of the propensity for crystal formation than measuring any single component in the urine [72].

## 8. How Can Physicians Help Their Patients to Increase Their Fluid Intake and Pay Attention to Its Composition?

### 8.1. Practical Ways to Increase Fluid Intake and Adherence to This Measure

Adequate fluid intake is a powerful and inexpensive strategy in urolithiasis prevention. Increased water intake for the prevention of urolithiasis recurrence has a significant cost-saving potential [75]. A sustained increase in urine volume was achieved over two decades in stone formers followed in a clinic dedicated to stone prevention, but the authors cannot document exactly how this was accomplished [76]. Patients can benefit from dietitian counseling and education on fluid intake. However, patients may have difficulties adhering to the suggested interventions. In order to increase patient compliance, stone formers should first be informed that they are at high risk of recurrence. Behavior change can be obtained if patients perceive the benefits of high fluid intake behind the constraint. There are many reasons why stone formers do not drink adequate amounts of fluids: water availability, aversion to the taste of water, feeling abdominal discomfort after ingesting high volume of fluids, lack of thirst, forgetting to drink water, and voiding frequency. An overactive bladder, bladder outlet obstruction, and voiding frequency incompatible with job activity such as, for example, professional drivers or schoolteachers, or limited access to bathrooms, are common barriers preventing an increased fluid intake [69,77,78].

Practical ways to increase fluid intake and daily routines should be advised such as drinking at set times during the day, drinking regularly despite lack of thirst, maintaining a water bottle in all places where significant time is spent, or carrying a water bottle and eating foods with a high content in water such as fruits and vegetables [69]. Self-monitoring using a simple urine color chart, a urine dipstick for urine specific gravity, or 24 h urine volume can help to succeed in increasing water intake [79,80,81]. Of note, 24 h urine collection should be performed preferably before each medical consultation to assess all risk factors for stone recurrence, including of course direct measurement of urine volume.

Digital tools and smart water bottles may also promote fluid consumption [82,83,84]; however, the effect on urine volume may be similar to counseling [85]. Smart water bottles that record fluid intake and associated digital applications, synced to the user’s smartphone, can provide fluid intake reminders and improve patients’ compliance [86]. Moreover, thanks to these connected tools, the fluid volume consumed can be accessible to physicians to evaluate their patients’ compliance.

Safety data on high fluid intake are limited [62]. Patients who eat little and drink a large volume may become overhydrated and subsequently hyponatremic while excreting maximally dilute urine, with water ingestion exceeding the kidney’s capacity for water excretion, as in the case of a “tea-and-toast” diet [87]. Urinary dilution can also be altered in the case of advanced renal failure [88] or with thiazide diuretic use [89], or in the case of a reduced effective circulating volume associated with non-osmotic mediated AVP release (e.g., congestive heart failure, cirrhosis) [87], leading to the risk of hyponatremia. In these patients, the blood sodium concentration should be closely monitored and advice regarding urine volume should be individualized and adjusted as necessary.

### 8.2. Advice Regarding Types of Fluid Intake

Fructose intake is associated with an increased risk of kidney stones [90] and urinary oxalate excretion increased after cola beverage consumption [91]. Consumption of sugar-sweetened soda [92,93], grapefruit juice [94], apple juice [94], and punch [92] is associated with a higher risk of stone formation, whereas consumption of coffee [92,94,95,96], tea [92,94], beer [92,94], and wine [92,94] is associated with a lower risk. 

Findings on the impact of orange juice intake on urinary risk factors for stone formation are inconsistent [97]. Curhan et al. reported no association between orange juice intake and the risk of stone formation [94] whereas Ferraro et al. reported a lower risk of stone formation with the consumption of orange juice [92]. Low-calorie orange juice can increase urinary citrate [98]. 

A meta-analysis has investigated the association between different types of beverages and risk of kidney stones [99]. Authors confirmed that increased water intake was associated with a reduced risk of developing kidney stones [99]. They also concluded that increased intake of coffee, tea, and alcohol showed potential benefits on stone prevention [99], although water remains the preferred fluid [12]. Patients should be advised to reduce their intake of sodas, in particular those acidified using phosphoric acid (typically colas) [93] and calorie-containing fluids and alcohol that may be detrimental to health [12]. Of note, black tea and green tea contain varying amounts of oxalate depending on the origin, quality, time of harvest, and preparation [97]. Consequently, tea should not be strongly steeped to avoid excessive oxalate intake. 

Concretely, physicians should advise their patients to preferentially drink water as it is free from calories, fructose, and alcohol. However, the composition of different mineral waters has to be taken into account since the mineral composition of bottled drinkable water and of tap water varies significantly [100,101,102,103,104], and may influence the urinary composition regarding stone promotors and inhibitors. 

Bicarbonate is a natural constituent of mineral water. Alkaline beverage will increase urinary pH and citrate excretion [105,106,107] so that drinking alkalizing beverages such as bicarbonate-rich mineral water can support alkalization therapy for uric acid and cystine stones while neutral pH beverages are recommended as general preventive measures for other type of stones [12]. Cystinuric patients are advised to maintain a urine pH between 7.5 and 8 in each freshly voided urine portion [12,14]. In case of stones containing uric acid, the targeted urine pH is 6.2–6.8 for prevention and 6.5–7.2 for chemolitholysis [12].

Calcium-rich mineral water can be used if the daily calcium intake achieved by consuming dairy products such as milk, cheese, or yoghurt is insufficient. Patients should be aware of the calcium content of the water and be advised to have a normal calcium diet, which is associated with a reduced risk of oxalate stone formation [67]. Importantly, calcium intake, whatever its source (food or beverage), must be spread over the three daily meals since intestinal calcium binds to oxalate at each meal, thereby decreasing its intestinal absorption and urinary excretion [108].

Finally, magnesium-rich mineral water can be used in the case of hypomagnesemia or of low urinary excretion of magnesium (<3 mmol/24 h [12]) related to poor dietary intake or to reduced intestinal absorption. Physicians should keep in mind that magnesium-rich mineral waters are most of the time also rich in calcium.

## 9. Pharmacotherapy to Prevent Nephrolithiasis

Other pharmacological interventions can be used to prevent nephrolithiasis such as thiazide diuretic in the case of persistent hypercalciuria after dietary measures in the absence of hypercalcemia and primary hyperparathyroidism [12]. Allopurinol or Febuxostat are warranted if elevated urinary excretion of uric acid persists after dietary measures and in the rare genetic disorder, 2,8-dihydroxyadeninuria [12,109]. Potassium citrate is prescribed in cases of alkalinization (e.g., cystinuria, uric acid calculi) or low urinary citrate excretion (e.g., distal tubular acidosis type 1, diarrhea) [12,14]. Magnesium is used in hypomagnesuria [12]. In refractory cystinuria, that is to say, if lithiasis occurs despite well-conducted alkaline hyperhydration, sulfhydryls (Tiopronin or D-penicillamine) are added [14]. Pyridoxine and novel RNA-interference-based therapy are used for managing patients with primary hyperoxaluria [15]. Despite these treatments, all patients are advised to maintain a high fluid intake to increase urine dilution of stone precursors.

## 10. Conclusions

In conclusion, increasing water intake to increase urine dilution is a recognized therapeutic approach to reduce the risk of kidney stones in all stone formers and represents the cornerstone of the preventive measures for all types of nephrolithiasis. Patients should be informed and supported in this difficult process of increasing urine dilution by increasing their fluid intake. This measure should be considered within a holistic approach to health. Further studies are needed on different types of stone formers including non-calcium stone formers and in patients at higher risk of developing kidney stones to determine the target of urine volume.

## Figures and Tables

**Table 1:** Fluid intake and urine volume goals in stone formers according to the type of nephrolithiasis and to the current guidelines.

	Fluid Amount	Diuresis	Urine Specific Gravity	Reference
**General preventive measures**				
European Association of Urology	2.5–3.0 L/day	2.0–2.5 L/day	<1.010	[12]
Canadian Urological Association		2.5 L/day		[69]
American College of Physicians		At least 2.0 L/day		[71]
American Urological Association		At least 2.5 L /day		[70]
**Cystinuria**				
European Association of Urology	3.5 L/day	>3 L/day		[12]
Canadian urological Association	3.5–4 L/day	>3 L/day		[69]
European Reference Network for Rare Kidney Diseases		>3 L/day	≤1.005	[14]
**Primary Hyperoxaluria**				
European Association of Urology	3.5–4.0 L/day			[12]
European Reference Network for Rare Kidney Diseases	3.5–4.0 L/day			[15]
**2,8-dihydroxyandenine stones and xanthine stones**				
European Association of Urology			<1.010	[12]

**Table 2:** Urinary concentration below which for a given lithogenic precursor, or above which for a given lithogenic inhibitor, the risk of crystallization is the lowest.

Urinary Parameters	Concentration	Reference
Calcium	<3.8 mmol/L	[60,74]
Oxalate	<0.31 mmol/L	[74]
Inorganic phosphate	<24 mmol/L at pH < 6.5	[73]
Urate	<2.4 mmol/L at 5.3 ≤ pH <5.5	[73]
	<2.8 mmol/L at 5.5 ≤ pH < 6	
	<3.5 mmol/L at pH ≥ 6	
Cystine	<1 mmol/L (250 mg/l)	[14]
Citrate	>1 mmol/L	[73]
Magnesium	>1.5 mmol/L	[73]

## Data Availability

Not applicable.
